# Understanding Cyber-Racism Perpetration within the Broader Context of Cyberbullying Theory: A Theoretical Integration

**DOI:** 10.3390/children10071156

**Published:** 2023-07-01

**Authors:** Jordan E. Scott, Christopher P. Barlett

**Affiliations:** Department of Psychological Sciences, Kansas State University, Manhattan, KS 66506, USA

**Keywords:** cyber-racism, cyberbullying, anonymity, online disinhibition

## Abstract

The purpose of the current theoretical review is to argue for the theoretical integration of cyber-racism perpetration into the broader cyberbullying context—making note of the similarities between both types of nefarious online behavior that make this integration appropriate and the differences that make the integration less clear. Cyber-racism and cyberbullying victimization have been shown to be prevalent in youth and is related to poor psychological outcomes. Understanding both types of antisocial online behaviors have implications for the understanding and subsequent reduction of cyber-racism. Our review focuses on a cyber-racism model that proposes the importance of anonymity perceptions afforded to the online user to cause cyber-racism via several routes that focus on (a) online disinhibition, (b) deindividuation and group polarization, and (c) stereotypes. We discuss the tenets of this theory and the overlap with the Barlett Gentile Cyberbullying Model—a learning-based model that focuses on how anonymity eventually predicts cyberbullying via the development of positive cyberbullying attitudes. We believe that theoretical integration is necessary; however, future work needs to test several theoretical underpinnings of these models first. We end with a discussion of theoretical and intervention implications before discussing limitations and future work. Overall, we hope this review sparks interesting future research to understand cyber-racism and broaden the existing research on cyberbullying.

## 1. Introduction

The Internet is ubiquitous. According to recent survey data, 97% of US teens use the Internet daily [1]. In today’s technologically savvy culture, today’s youth—more now than ever—use the Internet for various reasons and research has shown that the Internet can assist in identity development, aspirational development, and peer engagement [2]. For example, research has shown that frequency of Internet communication positively predicted friendship closeness to predict well-being in a sample of US teens (aged 10–17 years [3]). The Internet has allowed for near instantaneous access to information and communication across the world that has myriad positive applications for nearly every sector (e.g., medical, educational, financial, government). Despite these, and other, positive uses for the Internet, there are some who use the Internet to cause harm. The purpose of our current theoretical review is to focus on the similarities and differences in cyber-racism and cyberbullying perpetrations. The central question that we will address is whether cyberbullying and cyber-racism are conceptually and theoretically multicollinear or if both behaviors are different enough to address separately. To answer this question, we will first detail the paucity of research on cyber-racism—focusing on theory—before detailing the similarities and differences with the broader cyberbullying literature. The importance of understanding why and for whom cyber-racism and cyberbullying perpetration occur is paramount for intervention efforts aimed at reducing antisocial online behavior. Overall, we believe that this timely review will introduce, or at least highlight, the need for research devoted to the study of cyber-racism within the broader context of cyberbullying.

## 2. Cyber-Racism Perpetration: Definition, Research, and Theory

**Definition**. Cyber-racism is defined as “online communication that contains derogatory and harmful thoughts about racial supremacy and separation” [4] (p. 632), such as making harmful racial comments on social media, sharing information related to racial supremacy (e.g., White Nationalist information), and targeting others with harmful online attacks because of their race. Recent survey results show that 21% of Black US youth (aged 13–17) have personally experienced bullying/harassment online because of their race/ethnicity [5]. Another survey, which focused on Black US adults, showed that 25% have been harassed online due to their race/ethnicity [6]. Moreover, research has shown an increase in cyber-racism during the COVID-19 pandemic aimed at the Asian community [7]. In short, cyber-racism has emerged and has been perpetrated against multiple minority groups.

**Research**. Victims of cyber-racism experience several deleterious psychological outcomes, such as anxiety, depression moods, and low self-esteem [8,9]. Online racism has also been found to be related to psychological distress and increased alcohol use [10]. A key difference between more traditional forms of racism and cyber-racism that is important for our analysis is that the online nature of cyber-racism juxtaposed with the permanency of online communication creates a situation where a single instance of cyber-racism has the possibility of being shared (e.g., re-tweeting on Twitter), “liked”, forwarded, etc., and otherwise communicated multiple times. Therefore, from the victim’s point of view, one cyber-racism post can be compounded, which can be extremely harmful to one’s well-being. To protect oneself, people may feel the need to prepare themselves to face this type of racism, which ultimately leads to an anticipation of it happening. This anticipation can lead to hyper-vigilance in their life off the internet, which can be stressful and socially isolating [11,12].

**Theory.** Due to the detrimental effects victims of cyber-racism experience, it is vital that researchers focus on the mediating variables and processes that explain cyber-racism perpetration, and the variables that moderate those effects. Focusing on cyber-racism perpetration, and not victimization, allows for a better understanding for why—and for whom—cyber-racism perpetration is likely, which should assist in the development of interventions aimed at reducing cyber-racism. We believe (and hope) that, if such interventions are efficacious, reducing the likelihood of cyber-racism perpetration should reduce the number of cyber-racism victims—and their documented subsequent negative psychological outcomes. Currently, we are unaware of any published cyber-racism intervention. However, before scholars and intervention specialists delve into the creation of cyber-racism reduction curricula, validated theory derived from replicated empirical studies needs to be posited, tested, and evaluated. Fortunately, researchers have started to delve into the theoretical underpinnings of cyber-racism; however, a paucity of research has validated and tested them.

To our knowledge, there is only one proposed theory that focuses on cyber-racism perpetration: the Keum and Miller [13] model. This model consists of three routes to predict cyber-racism, which all differ in the proposed mediators. We do not believe that all three routes are mutually exclusive to each other—someone could engage in cyber-racism via one, two, or all three routes. All three routes start with anonymity perceptions, which is defined as the perceived increase in anonymity one has in an online environment [14,15]. Indeed, Lapidot-Lefler and Barak [16] described online anonymity as the condition of being unknown online including gender, age, location, and other personal details that could reveal one’s identity. Due to the perceived anonymity afforded to the online user, people who would not normally express racist ideas in an offline setting may be more likely to expound their prejudice ideals online if they believe their comments cannot be traced back to them [14]. In support of such claims, Keum [17] found that online users believe that (a) the Internet is an anonymous platform that allows people the confidence to express racism, and (b) online racism is a common occurrence and may be inevitable due to the greater accessibility of the Internet.

**Route 1**. The first route in the Keum and Miller [14] model suggests that online anonymity predicts cyber-racism through online disinhibition. In other words, online disinhibition mediates the relationship between anonymity and cyber-racism. The Online Disinhibition Effect [18,19] posits that people online may engage in various behaviors that they normally would not in the offline world. According to Suler [18], various facets of online disinhibition exist, which suggests multiple orthogonal processes that describe how behavior may differ in the online vs. offline world. These facets include (1) dissociative anonymity, defined as the perception that one’s identity can be hidden or changed when online, (2) invisibility, defined as social interactions occurring when people cannot physically see one another, (3) asynchronicity, defined as the gap in time between when harm is perpetrated to when the victim receives the harm, (4) solipsistic introjection, defined as the perceived image or voice of other people online which merges the real world with one’s online perceptions, (5) dissociative imagination, defined as the ability to psychologically escape or dissociate from online behaviors leading to the belief that the online world is imaginary and holds no connection to real life, and (6) minimization of status and authority, defined as the perceived absence or diminished views of real-life authority. Research has shown a significant positive correlation between anonymity perceptions and online disinhibition [20].

Udris [21] suggested that online disinhibition can be segmented into two subcategories: toxic and benign. Toxic online disinhibition consists of actions such as rude language, anger, hatred that people are more likely to exhibit online than in offline situations. Benign online disinhibition, on the other hand, describes acts of kindness and generosity [21]. The Keum and Miller [14] model focuses on toxic online disinhibition in its theorizing. Indeed, the model suggests that toxic online disinhibition mediates the direct relationship between anonymity perceptions and incidence of cyber-racism—the reason why anonymity perceptions are likely to predict cyber-racism is due to an increase in toxic online disinhibition.

We are unaware of any published literature examining the first route of the Keum and Miller [14] model; however, corollary evidence highlights the importance of online disinhibition and cyber-racism. For example, research focused on predicting online hate perpetration (actions directed at specific groups meant to harass, exclude, or promote violence towards [22]) found that toxic online disinhibition interacted with the perpetration of online hate such that when toxic online disinhibition was higher, online hate perpetration was higher as well. Moreover, Wachs and Wright [23] found that when higher online disinhibition was reported, victims of online hate themselves also reported higher levels of online hate perpetration. Less online hate perpetration was reported when online disinhibition was lower in the same study. Finally, findings from Wachs and Wright [23] showed a greater relationship between online hate victimization and perpetration when the individual was male, but not female. Another study found that when cyberbullying perpetration was reported, it was more likely that cyber-hate perpetration was also reported when there were higher levels of toxic online disinhibition [24].

**Route 2**. Keum and Miller [14] suggest that online anonymity can also affect deindividualization and theorize that this could lead to a higher likelihood of insensitivity, bias, and aggression towards racial differences. Deindividualization refers to the process in which a person loses their sense of self and their sense of individuality in social settings [25]. Keum and Miller [14] posit that deindividualization can lead to two additional paths: stereotyping and in-group bias. Both paths stem from the theory that individuals rely on their group (race/ethnicity) norms when interacting with others [26]. For instance, among White adults, 81% reported that all or most of their close friends were also white, and among Black adults, 70% reported that all or most of their close friends were black [27]. Therefore, people identify more with their group norms [28], and to demonstrate their group membership, individuals may be more likely to judge people outside of their group based on stereotypes [29,30].

Keum and Miller [14] theorized that greater group norm identification and stereotyping likely leads to the expression of cyber-racism. Research has shown that when our online interactions are anonymous and we cannot individuate who we are communicating with, we rely on our social stereotypes to interpret perceptions and behavior [31]. Another study found that when people were in a depersonalized condition, thus unidentifiable, their individual differences were less important, and they relied more on group membership and similarities [30]. Stereotyping is a strong predictor of prejudice attitudes [32,33], which can ultimately lead to someone expressing those thoughts online.

**Route 3**. The Keum and Miller [14] theory further posits that people have stronger identification within their group when relying on group norms—leading to in-group biases. In-group bias and out-group aversion can be predicted through stronger in-group identification [34]). On the other hand, when people can identify who they are interacting with and see them as a separate entity from a whole group, derogation, prejudice, and stereotyping are reduced [31]. Social dominance orientation [35] is one explanation for in-group bias and the aversion to outgroup members. Social dominance orientation is a personality trait that emphasizes that a social hierarchy is preferable in social settings, and thus, it is expected that higher status groups would dominate lower status groups [36]. Keum and Miller [14] also offers an explanation on why individuals with similar beliefs become a unified entity in online communities. Using results from Lee [37], which found that deindividuated people showed higher group identification and opinion polarization leading to higher in-group bias, Keum and Miller [14] noted that in-group bias could lead to group polarization. This polarization is when a group of like-minded people come together to share their ideas which in turn validates their opinions. This group solidarity can strengthen one’s opinions, in this case, those who share prejudicial attitudes and beliefs, and results in stronger confidence to share these ideas online versus offline. Group polarization has been found to be more likely in online situations than in face-to-face situations [38,39]. Tsuji and Kitamura [40] argued that online users who share similar attitudes and ideas communicate with each other and create closed networks where they only encounter ideas that affirm their beliefs. Once this group polarization occurs, we are not likely to change our minds about our opinions or attitudes towards certain ideas. Yardi and Boyd [41] found that in a group of Twitter responses to a polarizing event (i.e., the death of a doctor who performed abortions), when people of like-minded opinions replied to one another, their group identity was strengthened. In contrast, when opposite-minded individuals replied to one another, their in-group affiliation was strengthened as well as thoughts of out-group members. This study found that even when people are exposed to more viewpoints, meaningful discussion is limited [41].

## 3. Cyberbullying Perpetration: Definition, Research, and Theory

**Definition and Research.** Cyberbullying perpetration is defined as harming another individual, or group of individuals, repeatedly when the victims are motivated to avoid that harm using online or electronic means [42,43], and may include spreading false online rumors, online verbal attacks, and unwanted online sexual contact and sharing. Recent survey statistics show that 46% of US youth have been victimized online [5]. Extensive research has shown the deleterious psychological effects of both cyberbullying perpetration and victimization, including depression, anxiety, and low self-esteem [44].

**Theory**. We contend that theory is necessary to guide research aimed at understanding and, hopefully, reducing cyberbullying perpetration. Barlett [45] outlined the strengths and limitations of several social psychological, sociological, and communication theories that have been applied to predict cyberbullying—with great success. Indeed, research has shown that the tenets of the Theory of Planned Behavior [46,47], General Aggression Model [44,48], Uses-and-Gratifications Model [49,50], Social-Ecological Theory [51,52], Routine Activities Theory [53,54], and General Strain Theory [55,56] have all offered different lenses by which cyberbullying can be understood. Each theory offers a unique perspective on the predictors and processes involved in cyberbullying.

Each individual theory provides a different perspective by which to better understand cyberbullying perpetration, and each has their unique strengths and limitations. While it is not our position here to evaluate each theory, interested readers are directed to Barlett [45] for a comprehensive review. However, one important criticism of these theories is the inability to differentiate cyber from traditional bullying. Inspection of the tenets of any of these theories will reveal that the same variables and processes can be used to explain both types of bullying perpetration. For example, the Theory of Planned Behavior has been used to predict cyberbullying [47], traditional bullying [57], and aggressive behavior [58], and Social-Ecological Theory has been used to predict cyber [59] and traditional bullying [60]. While we contend that this does not diminish the contribution made to understanding cyberbullying, the ability for any theory to uniquely—or incrementally—predict cyberbullying beyond traditional bullying has important intervention implications. If theory can uncover the processes that uniquely predict cyberbullying, then bullying interventions can be adapted to cater specifically to both types of bullying simultaneously.

Research has suggested that cyber and traditional bullying are correlated [44], but differ in several important ways that make the transition from traditional to cyberbullying unclear. Scholars have contended that these two types of behaviors are different in several meaningful ways [61]. First, the online world necessitates that the interactions between the bully(ies) and victim(s) online are non-physical, and research has shown significant positive correlations between cyberbullying and verbal and relational types of aggression [62]. Second, users of the Internet may have increased perceptions of anonymity [18], which may lead to online disinhibition (defined and describe previously). Research has shown that online disinhibition predicts cyberbullying perpetration [21]. Third, because cyberbullying necessitates non-physical contact between the bully and victim, researchers have argued that one’s physical stature (e.g., muscularity and height) is no longer relevant to cyberbullying [63]; however, other forms of “power”, such as popularity [64], remain common across both types of bullying. Finally, the term “repeated” germane to the definition of traditional bullying likely manifests itself differently in online contexts. Indeed, one act of cyber-aggression perpetrated by one individual towards another online has the propensity to be shared, liked, and/or forwarded to others—thus, one act of cyber-harm may be repeated multiple times by others. Research has shown that cyberbullying perpetration correlates with the repeatability of online communications [65]. These, and perhaps additional, distinctions between cyber and traditional bullying perpetration are likely to have implications for interventions aimed at reducing cyberbullying. Again, we want to emphasize that the high meta-analytic correlation between both forms of bullying perpetration creates a likely intervention scenario in which traditional bullying intervention curricula can reduce both cyber and traditional bullying alike. For instance, ViSC is a traditional anti-bullying intervention that has been shown to reduce cyberbullying perpetration despite the lack of lessons unique to the online world [66]. However, interventions that emphasize the differences between cyber and traditional bullying are also successful. Barlett et al. [67] validated the You’re Not Anonymous (YNA) intervention to emphasize to participants that they are not anonymous online—any online communication (emails, text messages, messages sent via social media applications, social media posts, etc.) can be traced back to an individual device and/or user. Results showed that YNA participants had a decrease in anonymity perceptions and online disinhibition after the lessons were concluded, which predicted cyberbullying perpetration two months later.

We are aware of only one validated theory that can incrementally predict cyberbullying perpetration beyond traditional bullying by focusing on the previously elucidated differences between cyber and traditional bullying—the Barlett and Gentile Cyberbullying Model (BGCM [68]). The BGCM is a social-cognitive learning-based theory derived to uniquely predict cyberbullying perpetration. Inspired by the General Aggression [48] and General Learning [69] models, the BGCM posits that when an individual uses technology to harm someone for the first time, then they begin to believe in the irrelevance of muscularity for online bullying (BIMOB) and perceive themselves as anonymous—two knowledge structures that differentiate cyber from traditional bullying [61]. Continued cyber-aggressive behaviors further develop, and eventually automatize, these knowledge structures if these initial behaviors are positively reinforced. Consistent with Social Learning and Social Cognitive Theories [70], positive reinforcement strengthens the likelihood of behavior—including antisocial behaviors [71]. Research has shown that positive reinforcement for engaging in cyber-aggressive actions positively correlates with cyberbullying perpetration in a sample of US emerging adults [68], and punishment for similar behaviors negatively correlates with cyberbullying [72].

Each positively reinforced initial cyber-aggressive action serves as a learning trial in BGCM to automatize BIMOB and anonymity perceptions to eventually lead to the development of positive cyberbullying attitudes—the positive evaluation that cyberbullying is acceptable [73]. These positive attitudes are the immediate predictor of cyberbullying perpetration. Therefore, cyberbullying attitudes is the theoretical mediator in the relationship between (a) anonymity perceptions and cyberbullying perpetration and (b) BIMOB and cyberbullying perpetration. Support for the BGCM has been found in adult and youth populations [74]. Moreover, the tenets of BGCM have been found using correlational [68] and longitudinal [75] designs. Moreover, the BGCM has been shown to be valid cross-culturally. Using a cross-sectional research design, Barlett et al. [76] sampled participants from three independent countries (US, Germany, Australia) and four interdependent countries (Japan, China, Singapore, and Brazil), and found that the tenets of the BGCM were supported, but, more importantly, did not differ cross-culturally.

**Figure 1:**
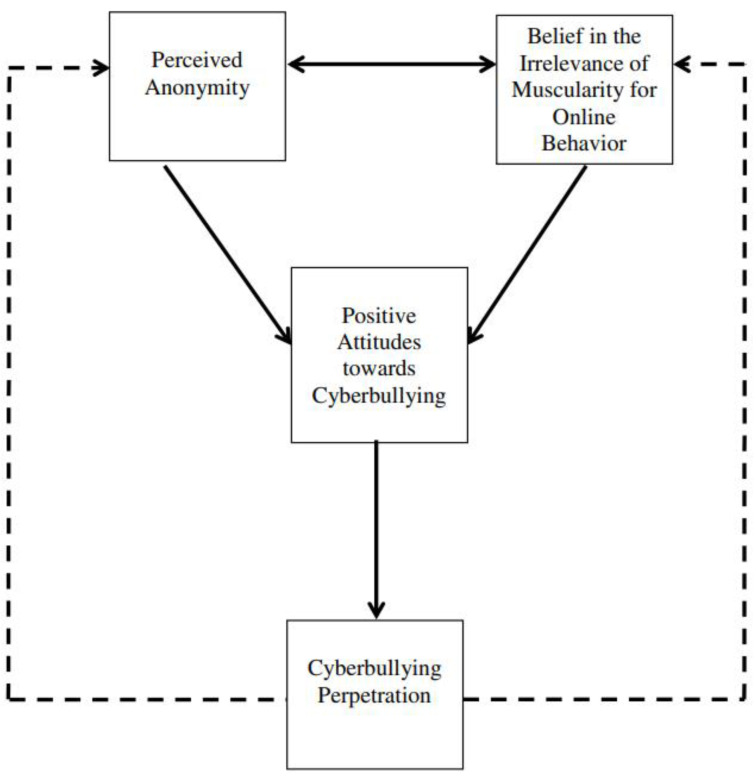
The Barlett Gentile Cyberbullying Model. Printed with Permission from Barlett, C.P. (2017). From theory to practice: Cyberbullying theory and its application to intervention. Computers in Human Behavior, 72, 271.

Finally, the BGCM incorporates a feedback loop, such that cyberbullying perpetration derived from the described BGCM processes further continues to reinforce the learned anonymity perceptions and BIMOB (see Figure 1). In a four-wave longitudinal study with US emerging adults, researchers found that Wave 1 anonymity and BIMOB predicted Wave 2 cyberbullying attitudes to predict Wave 3 cyberbullying perpetration—consistent with BGCM theorizing—and, moreover, Wave 3 cyberbullying perpetration positively predicted Wave 4 anonymity and BIMOB, showing the feedback loop [75]. Moreover, other longitudinal research showed that early cyberbullying attitudes and perpetrating behaviors predicted later cyberbullying attitudes and behaviors in a sample of Singaporean youth [77] and US youth [78].

The learning tenets of BGCM that focus on the feedback loop have implications for cyberbullying interventions. First, the ease and speed that cyberbullying knowledge structures (anonymity perceptions, BIMOB, and cyberbullying attitudes) may develop may present a challenge for cyberbullying prevention. Indeed, Gentile and Gentile [69] noted that the process of learning often takes time and experiences to shift from inexperienced to mastery. In the context of BGCM, this suggests that the act of engaging in a single cyber-aggressive action does not require much expertise or mastery of cyberbullying mechanics (how to do it), the intrinsic consequences (what the perpetrator learns or how the perpetrator feels after a cyber-aggressive incident), and the outcomes of the victim (the harm and ensuing psychological consequences the victim experiences). Continued cyber-aggression actions further reinforce these, and other consequences and outcomes that become learned and automatized to create a “cyberbully”. Second, the emphasis on reinforcement vs. punishment has implications for BGCM. Once an individual is reinforced for their cyber-aggressive behaviors, then the feedback loop is likely to continue, which further reinforces and automatizes cyberbullying knowledge structures to increase the likelihood of cyberbullying perpetration. Punishing initial cyber-aggressive behaviors, on the other hand, is likely to slow (or stop) the feedback loop to prevent cyberbullying learning. Thus, the role of parents, peers (bystanders), and school officials in successfully punishing or, at the very least, not positively reinforcing cyber-aggressive behaviors, is important.

## 4. Cyber-Racism and Cyberbullying: Theoretical Overlap and Differentiations

The central question posed at the beginning of our review addresses the degree of overlap between cyber-racism and cyberbullying perpetration. We will elaborate on two opposing arguments that differ in cyber-racism’s uniqueness.

**Similarity Viewpoint**. The first viewpoint posits that cyber-racism is cyberbullying that necessitates the harmful online content be focused on race/ethnicity. Implied in this viewpoint are at least two positions. First, the theoretical underpinnings of the BGCM—or any other theory focused on uniquely predicting cyberbullying—will be the same for cyber-racism. There is ample empirical and theoretical evidence to support this position. Indeed, examination of the Keum and Miller [14] cyber-racism model and the BGCM [68] will clearly show a focus on anonymity and online disinhibition in the prediction of cyber-racism and cyberbullying, respectfully, and that anonymity is located at the beginning of both models. These two theories both argue that anonymity perceptions do not directly predict their respective behavior, and both models rely on Suler’s [18] Online Disinhibition Effect to elucidate the reasoning behind the importance of anonymity for nefarious actions while online. Anonymity perceptions clearly represent an important exogenous variable. Second, if cyber-racism is an extension of cyberbullying, then one would expect cyber-racism frequency to be lower than cyberbullying. Indeed, if cyberbullying is broader, then there should be a smaller amount of harmful online content that focuses on race/ethnicity than more general harmful behaviors. Data support this claim—Vogels [5] showed that 21% of US youth have been victimized by cyber-racism and 46% of US teens are victims of cyberbullying. We do warrant caution in this comparison, because there are likely more White teens in these samples than any other race/ethnicity; however, given the lack of research addressing this question, these prevalence rates are the only method that exists currently to address this point.

Another key focus of similarity is the role of online disinhibition. Keum and Miller [14] noted that online disinhibition is a mediator in the relationship between anonymity and cyber-racism. The BGCM does not explicate the role of online disinhibition in cyberbullying prediction; however, it has been argued that online disinhibition is an important predictor of cyberbullying. Indeed, in a sample of US youth, researchers showed that toxic online disinhibition positively correlates with cyberbullying [79]—a finding that has been replicated across myriad other studies [22]; however, toxic online disinhibition was not a significant mediator in the relationship between anonymity perceptions and cyberbullying behavior.

Finally, the literature has shown personality correlates that influence cyberbullying perpetration, and should matter for cyber-racism; however, the extension of these personality variables to cyber-racism should be considered speculative until future empirical research can (dis)confirm these effects. We will discuss these in turn.

*Big 5*. The Big 5 represent stable personality traits [80] and consist of agreeableness (dependable, self-disciplined), openness (creative, nuanced), conscientiousness (organized, dependable), neuroticism (emotional unstable, critical), and extraversion (enthusiastic, social [81]). Several studies have examined the correlations between the Big 5 and cyberbullying; however, the findings vary greatly, likely due to characteristics of the sample (age of the participant, country the population was sampled), measurement differences between the studies, and other possible artifacts. Zhou et al. [82] showed that only agreeableness negatively correlated with cyberbullying, whereas Alonso and Romero [83] found that openness, conscientiousness, and agreeableness predicted cyberbullying, and Adamopoulou and Koukia [84] found that agreeableness and conscientiousness negatively correlated with cyberbullying perpetration. Currently, there is no published research that we are aware of that correlates the Big 5 with cyber-racism; however, there is vast research focused on the correlations between prejudice and the Big 5. Indeed, meta-analytic evidence suggests that extraversion (r = −0.07), agreeableness (r = −0.22), and openness (r = −0.30) negatively correlate with prejudice [85]. Because cyber-racism by definition, includes a prejudice aspect, we could theorize that these Big 5 variables should also correlate with cyber-racism.

*Dark Triad (Tetrad)*. The Dark Triad is a set of stable personality traits that represent one’s characterization of negative self-relevant attitudes, beliefs, and behaviors [86] and consists of narcissism (grandiose unstable sense of self), psychoticism (high callousness and high empathy), and Machiavellianism (manipulative and selfish [87]). Several research studies have shown that the Dark Triad correlates with cyberbullying perpetration [88,89,90,91]. Subsequent findings show that the Dark Triad is a better predictor of cyberbullying than the Big 5 [92]. As with the lack of research correlating the Big 5 to cyber-racism, there is no published research correlating the Dark Triad (or any individual constructs within the Dark Triad) to cyber-racism. However, results from the primary literature show that the Dark Triad positively correlates with prejudice [93,94,95]. Therefore, consistent with the Big 5, it is likely that the Dark Triad would predict cyber-racism; however, that is speculative.

*Other Personality Correlates*. Other personality variables have also been shown to correlate with cyberbullying perpetration and traditional forms of prejudice. For instance, research has shown that (a) moral disengagement correlates with cyberbullying [96] and prejudice [97], (b) empathy correlates with cyberbullying [98] and prejudice [99], and (c) sex differences have been observed for cyberbullying [100] and prejudice [101]. While this is not an exhaustive list of variables that correlate with both cyberbullying and prejudice, the point is that there are myriad variables that overlap with both forms of behavior that may (or may not) have implications for understanding and predicting cyber-racism.

**Differentiation Viewpoint**: We do not believe that anyone can argue the similarity viewpoint—especially the claim that cyber-racism is cyberbullying specific to harm targeting race/ethnicity. However, the differentiation viewpoint focuses more on the theoretical predictors of cyber-racism vs. cyberbullying perpetrations. There is ample theoretical reasoning and empirical data to suggest that predicting cyber-racism likely differs from cyberbullying despite the strong overlap between both types of harmful online behavior. First, the Keum and Miller [14] model offers two additional routes linking anonymity perceptions to cyber-racism that is absent from BGCM theorizing. These routes focus on the literature and theorizing from the intrapersonal bias and prejudice literatures. Having additional routes increases the psychological mechanisms that may explain cyber-racism that is largely absent from cyberbullying perpetration.

Second, there are personality variables that likely correlate with cyber-racism, but not cyberbullying perpetration more generally. Because cyber-racism blends the cyberbullying literature with the prejudice literature, it is likely that various moderating and mediating variables that are germane to cyber-racism processes are largely irrelevant to more general cyberbullying perpetration.

*Right-Wing Authoritarianism*. Right-wing authoritarianism (RWA) is a personality trait that consists of authoritarian submission (complete and unfettered domination to authority figures), conventionalism (undeterred adherence to conservative viewpoints), and authoritarian aggression (anger, hostility, and aggression aimed at those who violate traditional norms [102]). Findings across multiple empirical studies suggest that RWA is correlated with prejudice. For instance, Asbrock et al. [103] used a two-wave longitudinal design and showed that Wave 1 RWA predicted Wave 2 prejudice towards dangers groups and dissident groups. Moreover, participants who score high on RWA also score high on realistic and symbolic threat from outgroups [104], dangerous world beliefs [105], biased evaluations of mass media news reports [106], and high levels of patriotism and nationalism [107]. These results suggest that RWA is positively correlated with prejudice and the cognitive and motivational processes germane to prejudice and racism. Indeed, researchers asked adult participants to complete measures of the Big 5, RWA, SDO, and prejudice, and results from structural equation modeling analysis showed that RWA mediated the relationship between (a) conscientiousness and prejudice, (b) extraversion and prejudice, and (c) openness and prejudice; moreover, SDO mediated the relationship between agreeableness and prejudice [108]. Although we are unaware of any research focused on predicting cyber-racism from RWA, the aforementioned findings suggest that RWA should predict racism and cyber-racism; however, this is speculative and empirical investigation is desperately needed.

*Social Dominance Orientation*. Social dominance orientation (SDO) is a personality variable defined as one’s preference for inequality amongst groups [36]. Research has shown that individuals who score high on SDO also score high on (a) RWA [109], (b) nationalism and conservatism [110], in-group identification [35], and lack of empathy [111]. In addition, SDO is correlated with various prejudice outcomes [112]. Akin to the previous description of RWA, we are unaware of any study that correlates SDO with cyber-racism; however, we predict that SDO will be related to cyber-racism. Again, this is speculative and future work should examine this hypothesis.

Duckitt and Sibley [113] proposed a causal dual-process motivational model with two routes by which personality and the social context eventually lead to prejudice. In the first route, personality (e.g., low openness to experience) and a perceived threatening social situation leads to a dangerous worldview—the position that the world is a dangerous place—that predicts RWA. Finally, RWA predicts the perception that certain social interactions are threatening to predict increased prejudice. In the second route, personality (e.g., low agreeableness) and a competitive social situation leads to a worldview focused on resource competition that predicts SDO. Finally, SDO predicts the perception that competition and dominance is necessary to predict increased prejudice. Therefore, RWA and SDO are both mediators that are important process variables to eventually predict prejudice; however, RWA emphasizes the role of dangerous threats of an outgroup, whereas SDO focuses on perceived competition and dominance over an outgroup. Regardless of the specific route, SDO and RWA are important for prejudice. While we can only speculate on the direct or mediating roles of SDO and RWA on cyber-racism, the Duckitt and Sibley [113] theorizing is likely to translate to the online world. For instance, perhaps if an individual feels threatened, Keum and Miller [14] would argue that the anonymity afforded to the online user would provide an environment suitable to expound prejudicial beliefs and attitudes without fear of retaliation from authorities or the victim, which may be likely in real-world social interactions. Again, this is speculative, and future work should focus on these processes.

*Other Personality Variables*. RWA and SDO are two primary individual difference variables that have been shown to reliability predict prejudice and racism. As a result, we focused on those two variables; however, other personality variables unique to racism, but not bullying or aggression more broadly, may also highlight important differences between cyber-racism and cyberbullying perpetrations. Some include implicit biases [114], modern racism [115], and ambivalence [116]. These, and possibly other personality variables, may correlate with cyber-racism and/or moderate the relationship between other variables and cyber-racism. We do not view this list as comprehensive; however, these listed variables are only but a sample of other variables that are likely more relevant to cyber-racism than cyberbullying. Future work is desperately needed to test these speculative claims.

**Theoretical Compromise**. Thus far, we have presented evidence for both the theoretical fusion of cyberbullying and cyber-racism—which makes the most intuitive sense—and the differences that make integrating cyberbullying and cyber-racism less clear. We want to argue that perhaps a theoretical compromise would best describe the inter-relations between both types of antisocial behaviors: one that acknowledges the similarities between cyberbullying and cyber-racism while acknowledging and accounting for the differences. Given the paucity of research focused on cyber-racism perpetration, we cannot offer a new theoretical model that integrates the BGCM and the Keum and Miller [14] model together. However, there are some important considerations and comments that would be germane to such a model. We hope that these suggestions can provide insight and guide future research into examining cyber-racism prediction.

First, we must acknowledge that cyber-racism is cyberbullying specific to race/ethnicity. Therefore, all the predictors of cyberbullying should be pertinent to predict cyber-racism, such as anonymity, cyberbullying attitudes, BIMOB, and others—extensions of the BGCM to cyber-racism. The Keum and Miller [14] model already incorporates anonymity perceptions, providing the start of our theoretical compromise. Currently, there is no published research that correlates other aspects of BGCM to cyber-racism, such as BIMOB and cyberbullying attitudes, which should theoretically correlate. Finally, personality variables, such as the Big 5 and Dark Triad, may also correlate with cyber-racism to offer additional theoretical overlap with cyberbullying perpetration.

Second, we must also acknowledge that despite the theoretical overlap between cyberbullying and cyber-racism, there are key differences that make cyber-racism conceptually and theoretically different than cyberbullying perpetration. Indeed, there are variables and processes that are specific to the prejudice literature that should not correlate with more generalized bullying or cyberbullying that the study of cyber-racism can draw upon. Keum and Miller [14] already incorporate several routes that predict cyber-racism that emphasize stereotyping, in-group biases, deindividuation, and other processes. However, we also suggest that important personality variables, such as SDO and RWA, can explain additional variance in cyber-racism prediction; however, the Keum and Miller [14] model does not account for these variables.

Overall, we believe that a theoretical integration between Keum and Miller [14] and the BGCM is the best approach to predict cyber-racism; however, no research has been carried out to support these speculative claims. However, such integration seems intuitive. The shared theoretical overlap between both forms of nefarious online behaviors is clear when looking at each definition. Moreover, both theories already emphasize the role of anonymity, which creates a theoretical bridge connecting cyber-racism and cyberbullying perpetrations. From a theoretical point of view, the divergence in process between Keum and Miller [14] and the BGCM is interesting that requires an abundance of future research to understand. For example, how does BIMOB fit with the tenets of Keum and Miller [14]? We speculate that BIMOB will correlate with cyber-racism. Does route 2 of Keum and Miller [14]—the one focusing on deindividuation and in-group biases and polarization—matter for cyberbullying? We speculate that these processes will not predict cyberbullying unless the online harm is perpetrated onto an outgroup member. In short, the unique aspects of each theory that require separation juxtaposed with the shared processes between both theories needs empirical attention.

## 5. General Discussion

The purpose of the current theoretical review is to better understand the predictors and processes that predict cyber-racism perpetration. The majority of research thus far has focused on cyber-racism victimization, which has been shown to correlate with anxiety, depression, and low self-esteem [8,9]. However, the psychological processes that govern the decision to perpetrate racist communications online has primarily been theorized without much empirical data to support their postulates. Therefore, our goal was to enhance the understanding of cyber-racism perpetration, which has important implications for research, intervention and prevention efforts, and society who needs to better understand the various nefarious online behaviors youth may engage in.

From a theoretical perspective, we acknowledge that the processes derived from Keum and Miller [14] are (a) novel, (b) offer multiple routes to predict cyber-racism, and (c) offer a unique perspective that accounts for the likely high-statistical and conceptual relationship between traditional and cyber-racism juxtaposed with highlighted features that make online communication different than face-to-face communication. Indeed, the Keum and Miller [14] theory begins with the explication of the importance of anonymity afforded to the online user. We do want to explicate that traditional harm can be perpetrated against another in an anonymous manner—spreading rumors, verbal assaults, and even physical attacks can be carried out in anonymous forms; however, we contend that the online world provides the user with the perception of anonymity, and those perceptions are germane to harmful cyber-behaviors [45]. Vandebosch and Van Cleemput [61], and others have noted that anonymity perceptions are one key difference between cyber and traditional bullying perpetration, and research has shown that anonymity is significantly positively related to cyberbullying [14,15,117]. Barlett [45] was critical of theory that when applied to the online environment does not differentiate cyber from traditional harm, and one key strength of Keum and Miller [14] is that their theory begins with the explication of one of the key differences between online and real-world psychological processes. Moreover, Keum and Miller [14] proceed through their model by noting that online disinhibition predicts cyber-racism directly (route 1), which again satisfies the critique of Barlett [45]. Suler [18,19] noted how online communication provides a method by which individuals are more likely to engage in harmful behaviors (albeit online) than they would during face-to-face communication—as per the Online Disinhibition Effect (see also Udris [21]).

Finally, the Keum and Miller [14] model offers two additional routes that predict why anonymity eventually leads to cyber-racism. These other two routes are largely shared between the cyber and real world, as they focus on several variables, such as deindividuation perceptions, biases, in-group biases, stereotypes, and others. We are unaware of any research that has correlated cyber with more traditional methods of racism/prejudice/discrimination; however, we expect these two forms of racism to correlate highly. Therefore, the integration of computer-mediated communication variables (i.e., anonymity perceptions) combined with more traditional racism predictors and processes is welcomed, and, we argue, another key strength of the Keum and Miller [14] study.

While the Keum and Miller [14] model makes several interesting theoretical claims about cyber-racism prediction, we want to remind readers that we are unaware of any published empirical study testing the claims of this model. Despite this limitation and the desperate need for research to delve into the tenets of this model, we believe that an important first question that needs to be answered focuses on how cyber-racism is theoretically positioned within the broader cyberbullying perpetration literature and theorizing. Again, our conclusions are based on our theoretical understanding of both cyber-racism and cyberbullying perpetrations and should be considered speculative until research can (dis)confirm these claims. Relatedly, future work could also help elucidate the veridical nature of alternative perspectives. For instance, scholars could argue that the differentiation of cyber-racism and more traditional conceptualizations of racism is trivial, and, therefore, theory that explains the latter is sufficient. We hope that this paper argues against this perspective. Until published empirical findings that highlight the importance of accounting for the overlap between cyber and traditional racism while also acknowledging the key theoretical differences (i.e., online disinhibition, online anonymity) is conducted, this perspective is viable. We believe that the Keum and Miller [14] theory does accomplish this goal.

Our overall conclusion is that theoretical integration between the cyberbullying literature and the prejudice literature is necessary to increase the probability of predicting cyber-racism. Such integration is consistent with Keum and Miller [14]. Moreover, such integration will highlight the important similarities and differences that exist between cyber-racism and (a) cyberbullying and (b) traditional racism. We believe that cyber-racism is a special case of cyberbullying that focuses on racist communications. Therefore, the processes explicated by cyberbullying perpetration theory—derived from the BGCM—should also apply to cyber-racism. The shared importance of anonymity between BGCM and Keum and Miller [14] is a key example of such application. Moreover, myriad variables that have been shown to correlate with both prejudice and cyberbullying (e.g., Big 5, Dark Triad) should also correlate with cyber-racism—again suggesting shared components.

However, even with the degree of overlap hypothesized between cyberbullying and cyber-racism, there are key differences that likely make cyber-racism unique from other types of nefarious online behavior. For instance, there are key variables that predict racism (e.g., SDO, RWA) that should not influence cyberbullying perpetration, which draws more on the aggression and bullying literatures, but should predict cyber-racism. Moreover, the Keum and Miller [14] model explicates similar processes in routes 2 and 3.

In addition to basic theoretical knowledge regarding cyber-racism prediction, there are also several applied trajectories for the study of cyber-racism prediction. Given the wealth of literature that shows the deleterious psychological and behavioral effects of being cyber victimized and being discriminated against, intervention efforts aimed at reducing cyberbullying and racism are very important. Meta-analyses have shown the efficacy of several cyberbullying [118,119] and prejudice [120] reducing interventions, suggesting that these behaviors, attitudes, and beliefs can be changed. However, we are unaware of any research that has attempted to create, adapt, or validate an intervention targeted to reduce cyber-racism. Perhaps a parsimonious approach would be to include lessons about cyber-racism (or cyber-hate more generally) in an already existing cyberbullying or prejudice/bias intervention. Another approach would be to create a new intervention that specifically targets cyber-racism by combining efficacious intervention curricula. For example, Landazabal [121] designed an intense intervention for adolescent participants that focused on teaching (a) positive social behaviors, (b) conflict analysis and solutions, (c) intragroup communication and per acceptance, and (d) identify prejudices. Results showed that youth participants in the intervention condition had an increase in consideration of others, an increase in positive cognitions about outgroup members, and a decrease in negative cognitions about outgroup members compared to youth participants in the control group. These lessons could be integrated with cyberbullying interventions that focus on teaching how anonymity online is a myth [67] to reduce cyber-racism. Finally, there have been several meta-analytic reviews to suggest that intergroup contact can reduce prejudice because of an increase in empathy and a decrease in anxiety [122]. However, it is less clear whether intergroup contact can reduce cyber-racism, due to the influence of anonymity and online disinhibition in the Keum and Miller [14] model. Future work is needed to examine these issues. Regardless of how these lessons are crafted, we hypothesize that the best possible method to reduce cyber-racism is to build a curriculum built on solid theory derived from replicated empirical studies; however, consistent with Barlett [45], a theory unique to cyber-racism that combines the findings from the prejudice and cyberbullying literatures should yield an intervention tailored specifically to reduce cyber-racism.

Before concluding, we want to make note of some of the limitations of the Keum and Miller [14] model that necessitate future work and theorizing. Although we are strong supporters of Keum and Miller’s [14] model, we cannot state enough that empirical testing of parts of the model or the whole model are desperately needed. Indeed, the scientific method is situated to allow for data derived from theoretically-driven hypotheses to support or not support those hypotheses, which may beget alterations to theoretical tenets—if appropriate. While we note this limitation, we also acknowledge the challenge in studying these types of anti-social behaviors. Indeed, Barlett [45] noted the absence of a paradigm that validly measures actual (not hypothetical with vignettes) cyberbullying and cyber-racism. While great strides have been made regarding detection of cyberbullying via machine learning techniques of social media content [123], future work is needed to take these, or other, methods and apply them in a laboratory setting with participants who are actively typing, messaging, etc. Without these paradigms, then state-based cyberbullying and/or cyber-racism cannot be adequately studied—including some of the routes proposed by Keum and Miller [14]. Moreover, scientific attention is needed to examine whether the Keum and Miller [14] model is moderated. Is this model only applicable to White (or majority) participants? Does time spent online or technology access matter for this model’s postulates? Is one route stronger than the other at predicting cyber-racism? Do the tenets of this model remain while controlling for traditional racism? These and other questions need answers, but only after the initial Keum and Miller [14] model is supported with replicated data-driven findings.

## 6. Final Remarks

Cyber-racism is a major societal issue that is detrimental to the victim’s mental health. We believe that reducing cyber-racism perpetration through interventions will reduce subsequent cyber-racism victimization. There is currently little research and theory examining the processes and variables germane to cyber-racism perpetration, and we hope that this theoretical review will spurn myriad research ideas and empirical tests of the Keum and Miller [14]—or other—models that can ultimately help society better understand cyber-racism to inform intervention efforts.

## Data Availability

Not applicable.

## References

[1] Atske S. (2022). Teens, Social Media and Technology 2022, Pew Research Center: Internet, Science & Tech.

[2] Uhls Y., Ellison N., Subrahmanyam K. (2017). Benefits and costs of social media in adolescence. Pediatrics.

[3] Valkenburg P.M., Peter J. (2007). Internet communication and its relation to well-being: Identifying some underlying mechanisms. Media Psychol..

[4] Back L. (2002). Aryans reading Adorno: Cyber-culture and twenty-first century racism. Ethn. Racial Stud..

[5] Vogels E.A. (2022). Teens and Cyberbullying: 2022. https://www.pewresearch.org/internet/2022/12/15/teens-and-cyberbullying-2022/.

[6] Duggan M. (2017). 1 in 4 Black Americans Have Faced Online Harassment Because of Their Race or Ethnicity. www.pewresearch.org/fact-tank/2017/07/25/1-in-4-black-americans-have-faced-online-harassment-because-of-their-race-or-ethnicity.

[7] Dubey A. (2020). The Resurgence of cyber racism during the COVID-19 pandemic and its after effects: Analysis of sentiments and emotions in Tweets. JMIR Public Health Surveill..

[8] Tynes B.M., Giang M.T., Williams D.R., Thompson G.N. (2008). Online racial discrimination and psychological adjustment among adolescents. J. Adolesc. Health.

[9] Tynes B.M., Umaña-Taylor A.J., Rose C.A., Lin J., Anderson C.J. (2012). Online racial discrimination and the protective function of ethnic identity and self-esteem for African American adolescents. Dev. Psychol..

[10] Keum B.T., Ahn L.H. (2021). Impact of online racism on psychological distress and alcohol use severity: Testing ethnic-racial socialization and silence about race as moderators. Comput. Hum. Behav..

[11] Hickens M.T., Lee H., Hing A.K. (2018). The weight of racism: Vigilance and racial inequalities in weight-related measures. Soc. Sci. Med..

[12] Keum B.T., Li X. (2023). Online racism, rumination, and vigilance: Impact on distress, loneliness, and alcohol use. Couns. Psychol..

[13] Keum B.T., Miller M.J. (2018). Racism on the Internet: Conceptualization and recommendations for research. Psychol. Violence.

[14] Wright M.F. (2013). The relationship between young adults’ beliefs about anonymity and subsequent cyber aggression. Cyberpsychol. Behav. Soc. Netw..

[15] Wright M.F. (2014). Predictors of anonymous cyber aggression: The role of adolescents’ beliefs about anonymity, aggression, and the permanency of digital content. Cyberpsychol. Behav. Soc. Netw..

[16] Lapidot-Lefler N., Barak A. (2012). Effects of anonymity, invisibility, and lack of eye-contact on toxic online disinhibition. Comput. Hum. Behav..

[17] Keum B.T. (2017). Qualitative examination on the influences of the Internet on racism and its online manifestation. Int. J. Cyber Behav. Psychol. Learn..

[18] Suler J. (2004). The online disinhibition effect. Cyberpsychol. Behav..

[19] Suler J. (2005). The online disinhibition effect. Int. J. Appl. Psychoanal. Stud..

[20] Scott R.A., Stuart J., Barber B.L. (2022). What predicts online disinhibition? Examining perceptions of protection and control online and the moderating role of social anxiety. Cyberpsychol. Behav. Soc. Netw..

[21] Udris R. (2014). Cyberbullying among high school students in Japan: Development and validation of the Online Disinhibition Scale. Comput. Hum. Behav..

[22] Wachs S., Wright M.F. (2018). Associations between bystanders and perpetrators of online hate: The moderating role of toxic online disinhibition. Int. J. Environ. Res. Public Health.

[23] Wachs S., Wright M.F. (2019). The moderation of online disinhibition and sex on the relationship between online hate victimization and perpetration. Cyberpsychol. Behav. Soc. Netw..

[24] Wachs S., Wright M.F., Vazsonyi A.T. (2019). Understanding the overlap between cyberbullying and cyberhate perpetration: Moderating effects of toxic online disinhibition. Crim. Behav. Ment. Health.

[25] Festinger L., Pepitone A., Newcomb T. (1952). Some consequences of deindividuation in a group. J. Abnorm. Soc. Psychol..

[26] Postmes T., Spears R., Sakhel K., De Groot D. (2001). Social influence in computer-mediated communication: The effects of anonymity on group behavior. Personal. Soc. Psychol. Bull..

[27] Parker K., Menasce Horowitz J., Morin R., Hugo Lopez M. (2022). Chapter 5: Race and Social Connections-Friends, Family, and Neighborhoods. Pew Research Center’s Social & Demographic Trends Project. https://www.pewresearch.org/social-trends/2015/06/11/chapter-5-race-and-social-connections-friends-family-and-neighborhoods/.

[28] Reicher S.D., Spears R., Postmes T. (1995). A social identity model of deindividuation phenomena. Eur. Rev. Soc. Psychol..

[29] Douglas K.M., McGarty C. (2001). Identifiability and self-presentation: Computer-mediated communication and intergroup inter-action. Br. J. Soc. Psychol..

[30] Postmes T., Spears R., Lea M. (2002). Intergroup differentiation in computer-mediated communication: Effects of depersonalization. Group Dyn. Theory Res. Pract..

[31] Postmes T., Spears R., Lea M., Ellemers N., Spears R., Doosje B. (1999). Social identity, group norms, and “deindividuation”: Lessons from computer-mediated communication for social influence in the group. Social Identity: Context, Commitment, Content.

[32] Correll J., Park B., Judd C.M., Wittenbrink B. (2002). The police officer’s dilemma: Using ethnicity to disambiguate potentially threatening individuals. J. Personal. Soc. Psychol..

[33] Dovidio J.F., Gaertner S.L., Kawakami K., Hodson G. (2002). Why can’t we just get along? Interpersonal biases and interracial distrust. Cult. Divers. Ethn. Minor. Psychol..

[34] Hewstone M., Rubin M., Willis H. (2002). Intergroup bias. Annu. Rev. Psychol..

[35] Sidanius J., Pratto F., Mitchell M. (1994). In-group identification, social dominance orientation, and differential intergroup social allocation. J. Soc. Psychol..

[36] Pratto F., Sidanius J., Stallworth L.M., Malle B.F. (1994). Social dominance orientation: A personality variable predicting social and political attitudes. J. Personal. Soc. Psychol..

[37] Lee E.J. (2007). Deindividuation effects on group polarization in computer-mediated communication: The role of group identification, public-self-awareness, and perceived argument quality. J. Commun..

[38] Spears R., Lea M., Lee S. (1990). Deindividuation and group polarization in computer- mediated communication. Br. J. Soc. Psychol..

[39] Lataneé B., L’Herrou T. (1996). Spatial clustering in the conformity game: Dynamic social impact in electronic groups. J. Personal. Soc. Psychol..

[40] Tsuji D., Kitamura S. (2020). Exposure to online news and polarization of xenophobic attitudes: A quantitative analysis of survey data in Japan and the U.S. Osaka Hum. Sci..

[41] Yardi S., Boyd D. (2010). Dynamic debates: An analysis of group polarization over time on Twitter. Sage J..

[42] Englander E., Donnerstein E., Kowalski R., Lin C.A., Parti K. (2018). Defining cyberbullying. Pediatrics.

[43] Smith P.K., Mahdavi J., Carvalho M., Fisher S., Russell S., Tippett N. (2008). Cyberbullying: Its nature and impact in secondary school pupils. J. Child Psychol. Psychiatry.

[44] Kowalski R.M., Giumetti G.W., Schroeder A.N., Lattanner M.R. (2014). Bullying in the digital age: A critical review and meta-analysis of cyberbullying research among youth. Psychol. Bull..

[45] Barlett C.P. (2019). Predicting Cyberbullying: Research, Theory, and Intervention.

[46] Ajzen I. (1991). The theory of planned behavior. Organ. Behav. Hum. Decis. Process..

[47] Doane A.N., Pearson M.R., Kelley M.L. (2014). Predictors of cyberbullying perpetration among college students: An application of the theory of reasoned action. Comput. Hum. Behav..

[48] Anderson C.A., Bushman B.J. (2002). Human aggression. Annu. Rev. Psychol..

[49] Rubin A.M. (2009). Uses-and-gratifications perspective on media effects. Media Effects.

[50] Tanrikulu I., Erdur-Baker Ö. (2021). Motives behind cyberbullying perpetration: A test of uses and gratifications theory. J. Interpers. Violence.

[51] Bronfenbrenner U. (1994). Ecological models of human development. Int. Encycl. Educ..

[52] Cross D., Barnes A., Papageorgiou A., Hadwen K., Hearn L., Lester L. (2015). A social–ecological framework for understanding and reducing cyberbullying behaviours. Aggress. Violent Behav..

[53] Felson M. (2016). The routine activity approach. Environmental Criminology and Crime Analysis.

[54] Navarro J.N., Jasinski J.L. (2012). Going cyber: Using routine activities theory to predict cyberbullying experiences. Sociol. Spectr..

[55] Agnew R., White H.R. (1992). An empirical test of general strain theory. Criminology.

[56] Paez G.R. (2018). Cyberbullying among adolescents: A general strain theory perspective. J. Sch. Violence.

[57] Jaber L.S., Rinaldi C.M., Saunders C.D., Scott J. (2022). The intent behind bullying: An application and expansion of the Theory of Planned Behaviour. Contemp. Sch. Psychol..

[58] Finigan-Carr N.M., Cheng T.L., Gielen A., Haynie D.L., Simons-Morton B. (2015). Using the theory of planned behavior to predict aggression and weapons carrying in urban African American early adolescent youth. Health Educ. Behav..

[59] Guo S., Liu J., Wang J. (2021). Cyberbullying roles among adolescents: A social-ecological theory perspective. J. Sch. Violence.

[60] Merrin G.J., Espelage D.L., Hong J.S. (2018). Applying the social-ecological framework to understand the associations of bullying perpetration among high school students: A multilevel analysis. Psychol. Violence.

[61] Vandebosch H., Van Cleemput K. (2008). Defining cyberbullying: A qualitative research into the perceptions of youngsters. CyberPsychol. Behav..

[62] Johansson S., Englund G. (2021). Cyberbullying and its relationship with physical, verbal, and relational bullying: A structural equation modelling approach. Educ. Psychol..

[63] Barlett C.P., Prot S., Anderson C.A., Gentile D.A. (2017). An empirical examination of the strength differential hypothesis in cyberbullying behavior. Psychol. Violence.

[64] Wegge D., Vandebosch H., Eggermont S., Pabian S. (2016). Popularity through online harm: The longitudinal associations between cyberbullying and sociometric status in early adolescence. J. Early Adolesc..

[65] Davison C.B., Stein C.H. (2014). The dangers of cyberbullying. N. Am. J. Psychol..

[66] Gradinger P., Yanagida T., Strohmeier D., Spiel C. (2016). Effectiveness and sustainability of the ViSC social competence program to prevent cyberbullying and cyber-victimization: Class and individual level moderators. Aggress. Behav..

[67] Barlett C.B., Heath J.B., Madison C.M., DeWitt C.C., Kirkpatrick S.M. (2020). You’re not anonymous online: The development and validation of a new cyberbullying intervention curriculum. Psychol. Pop Media..

[68] Barlett C.P., Gentile D.A. (2012). Attacking others online: The formation of cyberbullying in late adolescence. Psychol. Pop. Media Cult..

[69] Gentile D.A., Gentile J.R. (2021). Learning from Video Games (and Everything Else): The General Learning Model.

[70] Bandura A. (1978). Social learning theory of aggression. J. Commun..

[71] Bandura A., Ross D., Ross S.A. (1963). Imitation of film-mediated aggressive models. J. Abnorm. Soc. Psychol..

[72] Hsieh M.L., Wang S.Y.K., Lin Y. (2023). Perceptions of punishment risks among youth: Can cyberbullying be deterred?. J. Sch. Violence.

[73] Barlett C.P., Helmstetter K., Gentile D.A. (2016). The development of a new cyberbullying attitude measure. Comput. Hum. Behav..

[74] Barlett C.P., Chamberlin K. (2017). Examining cyberbullying across the lifespan. Comput. Hum. Behav..

[75] Barlett C.P., Kowalewski D.A. (2019). Learning to cyberbully: An extension of the Barlett Gentile cyberbullying model. Psychol. Pop. Media Cult..

[76] Barlett C.P., Seyfert L.W., Simmers M.M., Chen V.H.H., Cavalcanti J.G., Krahé B., Suzuki K., Warburton W.A., Wong R.Y.M., Pimentel C.E. (2021). Cross-cultural similarities and differences in the theoretical predictors of cyberbullying perpetration: Results from a seven-country study. Aggress. Behav..

[77] Barlett C.P., Gentile D.A., Chng G., Li D., Chamberlin K. (2018). Social media use and cyberbullying perpetration: A longitudinal analysis. Violence Gend..

[78] Barlett C.P., Coyne S.M. (2023). Learning to cyberbully: Longitudinal relations between cyberbullying attitudes and perpetration and the moderating influence of participant sex: A brief report. Aggress. Behav..

[79] Wright M.F., Wachs S. (2021). Does empathy and toxic online disinhibition moderate the longitudinal association between witnessing and perpetrating homophobic cyberbullying?. Int. J. Bullying Prev..

[80] McCrae R.R., Costa P.T. (1989). Rotation to maximize the construct validity of factors in the NEO Personality Inventory. Multivar. Behav. Res..

[81] Gosling S.D., Rentfrow P.J., Swann W.B. (2003). A very brief measure of the Big-Five personality domains. J. Res. Personal..

[82] Zhou Y., Zheng W., Gao X. (2019). The relationship between the big five and cyberbullying among college students: The mediating effect of moral disengagement. Curr. Psychol..

[83] Alonso C., Romero E. (2017). Aggressors and victims in bullying and cyberbullying: A study of personality profiles using the five-factor model. Span. J. Psychol..

[84] Adamoposulou P., Koukia E. (2020). The effect of personality traits on the roles of traditional bully-victim and cyberbully–cybervictim among Greek adolescents. Int. J. Caring Sci..

[85] Sibley C.G., Duckitt J. (2008). Personality and prejudice: A meta-analysis and theoretical review. Personal. Soc. Psychol. Rev..

[86] Paulhus D.L., Williams K.M. (2002). The dark triad of personality: Narcissism, Machiavellianism, and psychopathy. J. Res. Personal..

[87] Jonason P.K., Webster G.D. (2010). The dirty dozen: A concise measure of the dark triad. Psychol. Assess..

[88] Goodboy A.K., Martin M.M. (2015). The personality profile of a cyberbully: Examining the Dark Triad. Comput. Hum. Behav..

[89] Safaria T., Lubabin F., Purwandari E., Ratnaningsih E.Z., Khairani M., Saputra N.E., Rahmawati E.I., Esita Z., Nazriani D., Miftahuddin M. (2020). The role of dark triad personality on cyberbullying: Is it still a problem?. Int. J. Sci. Technol. Res..

[90] Schade E.C., Voracek M., Tran U.S. (2021). The nexus of the dark triad personality traits with cyberbullying, empathy, and emotional intelligence: A structural-equation modeling approach. Front. Psychol..

[91] Wright M.F., Huang Z., Wachs S., Aoyama I., Kamble S., Soudi S., Li Z., Lei L., Shu C. (2020). Associations between cyberbullying perpetration and the dark triad of personality traits: The moderating effect of country of origin and gender. Asia Pac. J. Soc. Work. Dev..

[92] van Geel M., Goemans A., Toprak F., Vedder P. (2017). Which personality traits are related to traditional bullying and cyberbullying? A study with the Big Five, Dark Triad and sadism. Personal. Individ. Differ..

[93] Anderson J., Cheers C. (2018). Does the dark triad predict prejudice?: The role of machiavellianism, psychopathy, and narcissism in explaining negativity toward asylum seekers. Aust. Psychol..

[94] Hodson G., Hogg S.M., MacInnis C.C. (2009). The role of “dark personalities” (narcissism, Machiavellianism, psychopathy), Big Five personality factors, and ideology in explaining prejudice. J. Res. Personal..

[95] Koehn M.A., Jonason P.K., Davis M.D. (2019). A person-centered view of prejudice: The Big Five, Dark Triad, and prejudice. Personal. Individ. Differ..

[96] Lo Cricchio M.G., García-Poole C., te Brinke L.W., Bianchi D., Menesini E. (2021). Moral disengagement and cyberbullying involvement: A systematic review. Eur. J. Dev. Psychol..

[97] Camodeca M., Baiocco R., Posa O. (2019). Homophobic bullying and victimization among adolescents: The role of prejudice, moral disengagement, and sexual orientation. Eur. J. Dev. Psychol..

[98] Ang R.P., Goh D.H. (2010). Cyberbullying among adolescents: The role of affective and cognitive empathy, and gender. Child Psychiatry Hum. Dev..

[99] Miklikowska M. (2018). Empathy trumps prejudice: The longitudinal relation between empathy and anti-immigrant attitudes in adolescence. Dev. Psychol..

[100] Barlett C., Coyne S.M. (2014). A meta-analysis of sex differences in cyber-bullying behavior: The moderating role of age. Aggress. Behav..

[101] Jonason P.K., Underhill D., Navarrate C.D. (2020). Understanding prejudice in terms of approach tendencies: The Dark Triad traits, sex differences, and political personality traits. Personal. Individ. Differ..

[102] Altemeyer B. (1988). Enemies of Freedom: Understanding Right-Wing Authoritarianism.

[103] Asbrock F., Sibley C.G., Duckitt J. (2010). Right-wing authoritarianism and social dominance orientation and the dimensions of generalized prejudice: A longitudinal test. Eur. J. Personal..

[104] Caricati L., Mancini T., Marletta G. (2017). The role of ingroup threat and conservative ideologies on prejudice against immigrants in two samples of Italian adults. J. Soc. Psychol..

[105] Crowson H.M. (2009). Right-wing authoritarianism and social dominance orientation: As mediators of worldview beliefs on attitudes related to the war on terror. Soc. Psychol..

[106] Crawford J.T., Jussim L., Cain T.R., Cohen F. (2013). Right-wing authoritarianism and social dominance orientation differentially predict biased evaluations of media reports. J. Appl. Soc. Psychol..

[107] Osborne D., Milojev P., Sibley C.G. (2017). Authoritarianism and national identity: Examining the longitudinal effects of SDO and RWA on nationalism and patriotism. Personal. Soc. Psychol. Bull..

[108] Ekehammar B., Akrami N., Gylje M., Zakrisson I. (2004). What matters most to prejudice: Big five personality, social dominance orientation, or right-wing authoritarianism?. Eur. J. Personal..

[109] Whitley B.E. (1999). Right-wing authoritarianism, social dominance orientation, and prejudice. J. Personal. Soc. Psychol..

[110] Pratto F., Stallworth L.M., Conway-Lanz S. (1998). Social Dominance Orientation and the Ideological Legitimization of Social Policy 1. J. Appl. Soc. Psychol..

[111] Sidanius J., Kteily N., Sheehy-Skeffington J., Ho A.K., Sibley C., Duriez B. (2013). You’re inferior and not worth our concern: The interface between empathy and social dominance orientation. J. Personal..

[112] Ho A.K., Sidanius J., Pratto F., Levin S., Thomsen L., Kteily N., Sheehy-Skeffington J. (2012). Social dominance orientation: Revisiting the structure and function of a variable predicting social and political attitudes. Personal. Soc. Psychol. Bull..

[113] Duckitt J., Sibley C.G. (2009). A dual-process motivational model of ideology, politics, and prejudice. Psychol. Inq..

[114] Payne B.K., Hannay J.W. (2021). Implicit bias reflects systemic racism. Trends Cogn. Sci..

[115] McConahay J.B., Dovidio J.F., Gaertner S.L. (1986). Modern racism, ambivalence, and the Modern Racism Scale. Prejudice, Discrimination, and Racism.

[116] Markus H.R. (2008). Pride, prejudice, and ambivalence: Toward a unified theory of race and ethnicity. Am. Psychol..

[117] Barlett C.P. (2015). Predicting adolescent’s cyberbullying behavior: A longitudinal risk analysis. J. Adolesc..

[118] Gaffney H., Farrington D.P., Espelage D.L., Ttofi M.M. (2019). Are cyberbullying intervention and prevention programs effective? A systematic and meta-analytical review. Aggress. Violent Behav..

[119] Polanin J.R., Espelage D.L., Grotpeter J.K., Ingram K., Michaelson L., Spinney E., Valido A., Sheikh A.E., Torgal C., Robinson L. (2022). A systematic review and meta-analysis of interventions to decrease cyberbullying perpetration and victimization. Prev. Science..

[120] Paluck E.L., Green D.P. (2020). Cooperative Learning, Media, and Reading Interventions Show Promise in Reducing Prejudice. RRAPP.

[121] Landazabal M.G. (2002). Assessment of an intervention on social behaviour, intragroup relations, self-concept and prejudice cognitions during adolescence. Int. J. Psychol. Psychol. Ther..

[122] Pettigrew T.F., Tropp L.R. (2008). How does intergroup contact reduce prejudice? Meta-analytic tests of three mediators. Eur. J. Soc. Psychol..

[123] Van Hee C., Jacobs G., Emmery C., Desmet B., Lefever E., Verhoeven B., De Pauw G., Daelemans W., Hoste V. (2018). Automatic detection of cyberbullying in social media text. PLoS ONE.

